# Damage Identification Method for Medium- and Small-Span Bridges Based on Macro-Strain Data under Vehicle–Bridge Coupling

**DOI:** 10.3390/ma15031097

**Published:** 2022-01-30

**Authors:** Hao Zhang, Zhixin Zhong, Junmiao Duan, Jianke Yang, Zhichao Zheng, Guangxun Liu

**Affiliations:** 1 State Key Laboratory of Structural Mechanics Behavior and System Safety in Traffic Engineering, Shijiazhuang Tiedao University, Shijiazhuang 050043, China; 2 Structural Health Monitor and Control Institute, Shijiazhuang Tiedao University, Shijiazhuang 050043, China; 3 School of Civil Engineering, Shijiazhuang Tiedao University, Shijiazhuang 050043, China; zzxjgjkjc@foxmail.com (Z.Z.); yang18033702683@163.com (J.Y.); zhengzhichao0217@163.com (Z.Z.); liuguangxun0518@163.com (G.L.); 4 School of Transportation, Shijiazhuang Tiedao University, Shijiazhuang 050043, China; duanjunmiao@163.com

**Keywords:** macro-strain mode, medium- and small-span bridges, wavelet transform, cross-correlation function

## Abstract

The damage identification method based on macro-strain modality has shown good results for large-span flexible bridges. However, medium- and small-span bridges have a high stiffness, and the axle system is embodied. The strong time-varying vibration characteristics, coupled with the non-stationary characteristics of vehicle loads, make it difficult to accurately determine the stable strain modes of such bridges. To solve this problem, a damage localization index in the form of an amplitude vector matrix of the mutual energy density spectrum based on macro-strain was constructed using wavelet transform de-noising and reconstruction technology and cross-correlation function. The macro-static strain and macro-dynamic strain data obtained from a vehicle–bridge coupling experiment were reconstructed through wavelet transform, and the factors influencing the damage indices were analyzed. The results showed that the proposed indicators could help realize an accurate damage localization for medium- and small-span bridge systems with different damage degrees under the action of vehicle–bridge coupling.

## 1. Introduction

Transportation is vital to regional economic development, and bridges are key to maintaining a smooth traffic flow. The normal operation of a transportation system relies heavily on the health of bridge structures [1,2]. To ensure the safety of bridge operation, several health monitoring systems have been established. However, existing monitoring systems are complex, expensive, and mostly applicable to long-span bridges. In highway and railway bridges, the vast majority of bridges are medium- and small-span bridges (the middle bridge generally has the total length of porous span between 30 m and 100 m or the single span between 20 m and 40 m, and the small bridge mostly refers to the total length of porous span between 8 m and 30 m or the single span between 5 m and 20 m). The medium- and small-span bridges have small span and large stiffness, so the vehicle–bridge coupling effect is obvious. At the same time, small- and medium-span bridges are prone to cracks and reduce the structural stiffness of bridges. However, because of its complex structure and soft structure, long-span bridges are often the key objects of structural health monitoring and detection, while small- and medium-span bridges lack attention. In recent years, due to the increase in vehicle load, structural deterioration, and structural form that do not conform to the current specification, the tilting erosion and fracture of medium- and small-span bridges have occurred. Throughout the domestic and foreign bridge damage accident cases, the proportion of small- and medium-span bridge damage accidents accounted for the vast majority. Therefore, it is necessary to improve the real-time monitoring of the damage condition of medium- and small-span bridges, and develop a simple damage identification technology suitable for practical applications.

With the development of bridge construction and health monitoring technology, the damage identification method of bridge structure has developed rapidly. So far, many mature theoretical methods have emerged. The classification of monitoring data based on damage identification can be divided into a damage identification method based on static measurement data and a damage identification method based on dynamic monitoring data. The static-based damage identification method is reliable, but it is time-consuming, laborious, and expensive to interrupt the traffic in the detection, which is not conducive to the real-time evaluation of the bridge state. For example, static load test, percussion method, and millimeter wave radar method [3] based on non-destructive damage detection need to interrupt traffic for damage identification, which brings great inconvenience to people’s travel. However, the damage identification method based on dynamic response can not interrupt the traffic, and only relying on environmental incentives can achieve the goal of online damage identification for bridges. Different from acceleration sensors, long-gauge strain sensors can not only reflect the overall information of the bridge, but also represent the local information of the structure. The strain index is more sensitive to local damage [4], whereas the displacement index is more effective for overall damage identification [5]. The sensitivity for local damage identification is arranged from low to high, and local damage in structures can be more easily identified using the strain index than the displacement index [6]. With the development of macro-strain measurement technology, the distributed strain sensing technology realizes the regional sensing, which overcomes the shortcomings of the traditional ‘point’ sensing that is too local, and overcomes the shortcomings of the traditional displacement sensing and acceleration sensing that are too macroscopic. It integrates the local and global information of structures, enables dynamic and static testing, and has advantages such as accurate damage localization and high quantitative accuracy [7,8,9,10]. Therefore, in this paper, the measured macro-strain data will be used as the data source for medium- and small-span bridge damage identification.

Based on macro-strain data, the following damage identification methods have been developed in recent years. Li et al. [11,12] employed a macro-strain mode vector with a long gauge as the damage index; it exhibited a high accuracy for damage localization and quantification with a good application effect for long-span flexible bridges. Wu et al. [8] proposed a structural damage identification method based on macro-strain modal vector. The modal parameters were extracted by strain response, and then the two-level strategy for flexible structure damage detection was proposed. Hong [13] proposed a method of only output modal macro-strain extraction and a bridge damage identification method under environmental excitation, and theoretically proved that the modal macro-strain of the strain sensor was uniquely determined by the peak value of the dynamic macro-strain response power spectrum density (PSD). Then, this method was applied to the state assessment of a practical bridge in New Jersey. Xu et al. [14,15] proposed a damage identification method for long-span cable-stayed bridges based on the residual trend of the macro-strain modal and the energy of macro-strain frequency response function. This method is clear in theory and easy to implement. According to the mapping relationship between long strain and displacement, Zhang et al. [10] deduced the macro-strain frequency response function, and proposed two structural strain modal identification methods based on it, and verified the effectiveness of the two methods. Anastasopoulos [16] identified the strain mode and characteristic frequency of the prestressed concrete beam model by macro-strain data to identify the damage and damage location of the structure. There are obvious changes in the amplitude and curvature of the strain mode in the damage area. However, medium- and small-span bridges have high rigidity. When the vehicle-to-bridge mass ratio is relatively high, the vibration effect due to vehicle–bridge coupling cannot be ignored. The structural system will reflect the time-varying vibration characteristics, and the non-stationary characteristics of the vehicle load will make it difficult to accurately obtain the stable strain mode of the bridge [15,17]. Clearly, identifying damages in a time-varying bridge based on the macro-strain modal test method is challenging, and the existing macro-strain theory cannot be directly applied to the damage identification of medium- and small-span bridges [18,19]. Hong et al. [4] established a static damage identification method based on the difference of macro-strain influence line area before and after structural damage by using a vehicle moving load. Li [20] proposed a damage identification method for urban road viaducts based on the working deformation of the macro-strain in the frequency-domain. This method can accurately identify damage locations and provides a good solution to the macro-strain problems encountered in the damage identification of medium- and small-span bridges. The idea is to advance the application of macro-strain-based damage identification to medium- and small-span bridges. Razavi and Hadidi [21] proposed a structural damage identification method based on finite element model correction and wavelet packet transform component energy, and verified the effectiveness and applicability of this method in structural damage identification by taking a two-dimensional steel truss structure as an example. The results show that this method can accurately determine the existence, location, and magnitude of damage, but the method requires the finite element model of the structure without damage. The performance of damage identification using macro-strain data is excellent, and the vehicle–bridge ratio of medium- and small-span bridges is large. When a vehicle passes the bridge, obvious macro-strain data can be measured, which provides a guarantee for subsequent damage identification. However, the complicated damage identification theory makes it challenging for ordinary engineers to grasp. Therefore, how to make the damage identification method based on macro-strain more easily applied in the damage identification of medium- and small-span bridges has become a research focus.

As a time–frequency analysis method, wavelet analysis has the characteristics of multiresolution and has a strong ability to represent the local characteristics of a signal, particularly when dealing with non-stationary signals. It can realize an effective decomposition and noise reduction of signal data [22]. Yu [23] conducted a modal analysis of a cracked bridge based on the structural dynamic equation of motion, studied the modal characteristics of the damaged bridge, and realized damage localization through the wavelet coefficients of the wavelet transform. Liu [24] employed the curvature of the displacement response and the cross-correlation function of the vehicle-excited bridge response as damage indices and applied the time–frequency analysis method of lifting wavelet transforms to identify bridge damage under moving loads. This method does not require damage-free structural response information, and the damage can simply be identified through the vibration response data of the bridge under different moving loads. Although it can effectively identify the damage location, the degree of damage cannot be easily quantified. Macro-strain damage identification methods are mostly based on empirical mode decomposition analysis, while wavelet transform for noise reduction and time–frequency analysis are rarely used. 

Based on the above research, this paper reports a damage localization index in the form of an amplitude vector matrix of the mutual energy density spectrum based on macro-strain, constructed using wavelet transform de-noising and reconstruction technology and cross-correlation function. With the macro-dynamic strain and macro-static strain experimental data, the accuracy of the proposed damage localization index was verified in terms of the damage degree, damage location, and vehicle-to-bridge mass ratio. The experimental results showed that the proposed indicators could achieve precise damage localization for medium- and small-span bridge systems with different damage degrees under the action of vehicle–bridge coupling.

## 2. Theoretical Basis

### 2.1. Damage Identification Based on Vehicle–Bridge Coupling Theory

**Figure 1:**
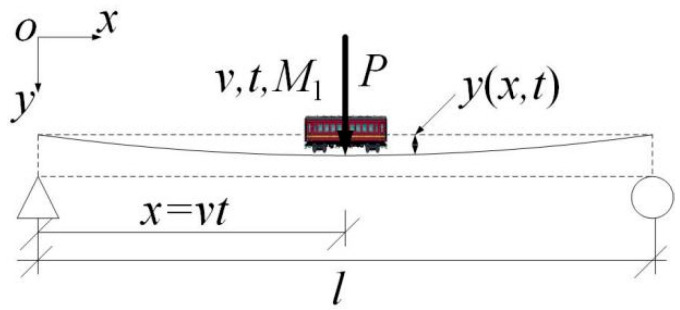
Vehicle–bridge time-varying system.

For a small-span bridge with a relatively high vehicle-to-bridge mass ratio, the moving vehicle-mounted action can be approximately considered vehicle–bridge coupling, and the vehicle–bridge system has time-varying characteristics [25,26,27]. This is shown in Figure 1. P is the force actions on the bridge; t is the vehicle travel time in the bridge; v is the speed of the vehicle; $M_{1}$ is the vehicle mass; y(x,t) is the vertical dynamic displacement of the bridge; x is the distance traveled by the vehicle on the bridge; l is the length of the bridge.

When the vehicle–bridge coupling effect is considered, the macro-strain generated by the moving vehicle-mounted action includes two categories. One is the macro-strain generated by the vehicle weight, i.e., the static strain. The other is the macro-dynamic strain due to the vehicle–bridge coupling. Therefore, based on the measured macro-strain data and wavelet transform, this study reconstructed and separated the macro-dynamic and static strains, and then used the reconstructed macro-static and dynamic strain data as the research object to perform an impact analysis on damage localization [19,28].

**Figure 2:**
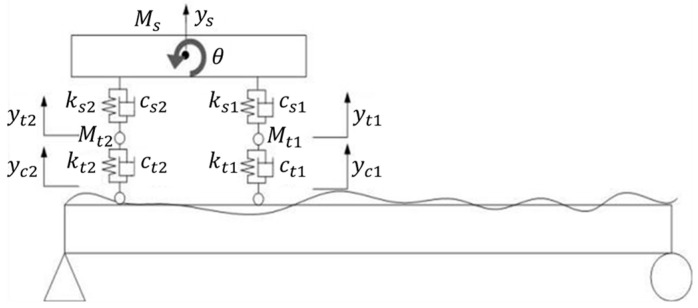
Coupling effect of the vehicle and bridge.

Based on the modal superposition method and D’Alembert’s principle, a vehicle–bridge coupling model was established [29], as shown in Figure 2. $M_{s}$ is the mass of the car body; $M_{t1}$ represents the total mass of suspension device of vehicle front axle and tires; $M_{t2}$ represents the total mass of rear axle suspension device and tires. $k_{s1}$ and $k_{s2}$ represent the spring stiffness of the front and rear axle suspension system of vehicle, respectively; $k_{t1}$ and $k_{t2}$ are, respectively, the stiffness of the front and rear axle tires of the vehicle. $c_{s1}$ and $c_{t1}$ are the damping of vehicle front axle suspension system and tires, respectively. On the contrary, $c_{s2}$ and $c_{t2}$ are the damping of vehicle rear axle suspension system and tires, respectively. $y_{s}$ is the vertical displacement of the car body. θ represents the rotation angle of the car body. $y_{t1}$ is the vertical displacement of vehicle front axle suspension system; $y_{t2}$ is the vertical displacement of vehicle rear axle suspension system; $y_{c1}$ and $y_{c2}$ are the vertical displacements of the front and rear axle tires, respectively.

The bridge vibration equations of the vehicle–bridge coupled vibration model shown in Figure 2 can be expressed as:

(1) $$[M_{v}]\left\{{\ddot{y}}_{v}\right\}+[C_{v}]\left\{{\dot{y}}_{v}\right\}+[K_{v}]\left\{y_{v}\right\}=\left\{F_{v-b}\right\}+\left\{F_{G}\right\}$$

(2) $$[M_{b}]\left\{{\ddot{y}}_{b}\right\}+[C_{b}]\left\{{\dot{y}}_{b}\right\}+[K_{b}]\left\{y_{b}\right\}=\left\{F_{b-v}\right\}$$

where $\left[M_{v}\right]$, $\left[C_{v}\right]$, and $\left[K_{v}\right]$ are the mass, damping, and stiffness matrices of the vehicle, respectively; $\left[M_{b}\right]$, $\left[C_{b}\right]$, and $\left[K_{b}\right]$ are, respectively, the mass, damping, and stiffness matrices of the bridge; $\left\{y_{b}\right\}$ and $\left\{y_{v}\right\}$ are the displacement vectors of the bridge and vehicle, respectively; $\left\{F_{v-b}\right\}$ and $\left\{F_{b-v}\right\}$ are, respectively, the combined force components of the vehicle–bridge coupling acting on the vehicle and bridge; $\left\{F_{G}\right\}$ is the gravity vector acting on the vehicle.

Based on the dynamic balance and displacement coordination relationship, the following matrix can be established:

(3) $$[\begin{array}{l} M_{v} \end{array}\begin{array}{l} M_{b} \end{array}]\left\{\begin{array}{l} {\ddot{y}}_{v}\\ {\ddot{y}}_{b} \end{array}\right\}+[\begin{array}{l} C_{v}\\ C_{b-v} \end{array}\begin{array}{l} C_{v-b}\\ C_{b}+C_{b-b} \end{array}]\left\{\begin{array}{l} {\dot{y}}_{v}\\ {\dot{y}}_{b} \end{array}\right\}+[\begin{array}{l} K_{v}\\ K_{b-v} \end{array}\begin{array}{l} K_{v-b}\\ K_{b}+K_{b-b} \end{array}]\left\{\begin{array}{l} y_{v}\\ y_{b} \end{array}\right\}=\left\{\begin{array}{l} F_{v-b}+F_{G}\\ F_{b-v} \end{array}\right\}$$

In this formula, $C_{v-b}$, $C_{b-v}$, $C_{b-b}$, $K_{v-b}$, $K_{b-v}$, $K_{b-b}$, $F_{v-b}$, and $F_{b-v}$ are the coupling terms generated by vehicle–bridge coupling. Generally, Newmark−β is used to solve the above coupling equation.

After solving for the displacement $y_{b}$ at each point in the bridge using this method, the angular displacement $\varphi_{b}$ of the point can be obtained by differentiating the displacement [30].

(4) $$\varphi_{b}(x,t)=-\frac{dy_{b}(x,t)}{dx}$$

(5) $$\bar{\varepsilon }(L_{m},t)=\frac{\varphi_{b}(x,t)-\varphi_{a}(x,t)}{L_{m}}h_{m}$$

where $\bar{\varepsilon }$ is the theoretical macro-strain, $L_{m}$ is the gauge length, $h_{m}$ is the height of the neutral axis. $\varphi_{a}(x,t)$ is the angular displacement of the point *a*, $\varphi_{b}(x,t)$ is the angular displacement of the point *b*.

### 2.2. Reconstruction of Macro-Strain by Wavelet Transform

The measured macro-dynamic response ${\bar{\varepsilon }}^{\prime }(L_{m},t)$ of the bridge under environmental excitation and moving load excitation includes complex response signals, such as the vehicle load and environmental excitation [31,32], as expressed in Equation (6). Therefore, it is necessary to eliminate the influence of environmental excitation, such as noise, and extract useful strain signals of vehicle load from the complex macro-dynamic strain signals, so that the time-varying bridge damage can be effectively identified.

(6) $${\bar{\varepsilon }}^{\prime }(L_{m},t)=\bar{\varepsilon }(L_{m},t)+\xi (t)$$

where, ${\bar{\varepsilon }}^{\prime }(L_{m},t)$ is the actual measured macro-strain, ξ(t) is the macro-strain response signal generated by environmental excitation.

In the actual engineering signal acquisition, the signal mostly contains many mutations and spikes, and the noise signal is not a stationary white noise signal. Therefore, when de-noising this non-stationary signal, the traditional Fourier transform cannot give the mutation of the signal at a certain time point, and it is difficult to effectively distinguish the mutation of the signal in the time domain, which makes it difficult to realize the accurate de-noising of non-stationary signals by Fourier transform. However, the wavelet transform is different. It can conduct signal analysis in both time and frequency domains at the same time, which can effectively distinguish the noise part of the signal and the mutation of the signal on the time axis, so as to complete the reasonable noise reduction of non-stationary signals. In the following, the measured macro-strain ${\bar{\varepsilon }}^{\prime }(L_{m},t)$ is decomposed and reconstructed by wavelet transform to remove the influence of environmental noise on macro-strain.

(7) $${\bar{\varepsilon }}^{\prime }(L_{m},t)=c_{1}A_{1}+c_{1}D_{1}+c_{2}A_{2}+c_{2}D_{2}\cdots c_{i}D_{i}$$

where i is the number of decomposition layers, $c_{i}A_{i}$ is the approximate part of the decomposition, $c_{i}D_{i}$ is the detailed part of the decomposition, and the noise part is typically included in $c_{i}D_{i}$. The de-noise separation can be applied to obtain the reconstructed macro-strain ${\bar{\varepsilon }}_{cg}(L_{m},t)$, as expressed in Equation (8).

(8) $${\bar{\varepsilon }}_{cg}(L_{m},t)=c_{1}A_{1}+c_{2}A_{2}+\cdots c_{i}A_{i}$$

## 3. Construction of Damage Indices

### Damage Localization Index Based on Macro-Strain

The reconstructed macro-strain response curve ${\bar{\varepsilon }}_{cg}(L_{m},t)$ can obtain ${\bar{\varepsilon }}_{cg}(L_{m},\omega )$ by Fourier transformed.

(9) $${\bar{\varepsilon }}_{cg}(L_{m},\omega )=\int_{-\infty }^{\infty }\psi (L_{m},t)e^{-j\omega t}dt=Re(\omega )+jIm(\omega )=|X\left(\omega \right)|e^{j\phi (\omega )}$$

where, the amplitude is $|X\left(\omega \right)|=\sqrt{Re^{2}(\omega )+Im^{2}(\omega )}$. Re(ω) and Im(ω) are, respectively, the real and imaginary parts of the Fourier function.

The macro-strain amplitude after Fourier transform was selected to perform a cross-correlation function calculation among the elements, and the mutual energy density spectrum of the macro-strain amplitude corresponding to the frequency $\omega =\frac{1}{t}$ was obtained, as expressed in Equation (10).

(10) $$m_{(n-1)\times n,\omega }^{\omega_{\omega }}=X(\omega_{n-1}^{\omega_{\omega }})\times X(\omega_{n}^{\omega_{\omega }})$$

where $\left|X(\omega_{n}^{\omega_{\omega }})\right|$ and $\left|X(\omega_{n-1}^{\omega_{\omega }})\right|$ are the macro-strain response amplitudes at the frequency of the measuring points of elements n and n−1, respectively.

The amplitude matrix of mutual energy density spectral between n elements in full frequency domain (or full-time domain) is obtained by calculating the cross-correlation function of the measured data of n long-gauge elements. The cross-correlation of a single element is self-energy density spectrum, and that of different elements is mutual energy density spectrum. The amplitude matrix has multiple forms. Therefore, to better describe its dynamic characteristics, all the types of deformations are uniformly named as the element energy product among the cross-correlated long-gauge elements in the frequency domain. This product is hereinafter referred to as the cross-correlation element energy product, which is equivalent to the amplitude vector of the macro-strain mutual energy density spectrum among the long-gauge elements.

(11) $$\left\{\begin{array}{cccccccccc} m_{1,1}^{\omega_{1}} & m_{1,2}^{\omega_{2}} & \cdots & m_{1,\omega -1}^{\omega_{\omega -1}} & m_{1,\omega }^{\omega_{\omega }} & m_{1,\omega +1}^{\omega_{1}} & m_{1,\omega +2}^{\omega_{2}} & \cdots & m_{1,2\omega }^{\omega_{\omega -1}} & m_{1,2\omega }^{\omega_{\omega }}\\ m_{2,1}^{\omega_{1}} & m_{2,2}^{\omega_{2}} & \cdots & m_{2,\omega -1}^{\omega_{\omega -1}} & m_{2,\omega }^{\omega_{\omega }} & m_{2,\omega +1}^{\omega_{1}} & m_{2,\omega +2}^{\omega_{2}} & \cdots & m_{2,2\omega }^{\omega_{\omega -1}} & m_{2,2\omega }^{\omega_{\omega }}\\ \vdots & \vdots & \vdots & \vdots & \vdots & \vdots & \vdots & \vdots & \vdots & \vdots \\ m_{(n-1)\times n,1}^{\omega_{1}} & \cdots & \cdots & \cdots & m_{(n-1)\times n,\omega }^{\omega_{\omega }} & m_{(n-1)\times n,\omega +1}^{\omega_{1}} & \cdots & \cdots & \cdots & m_{(n-1)\times n,2\omega }^{\omega_{\omega }}\\ m_{n\times n,1}^{\omega_{1}} & \cdots & \cdots & \cdots & m_{n\times n,\omega }^{\omega_{\omega }} & m_{n\times n,\omega +1}^{\omega_{1}} & \cdots & \cdots & \cdots & m_{n\times n,2\omega }^{\omega_{\omega }} \end{array}\right\}$$

where n∈Z, and the full-frequency domain is $\{\omega_{1},\omega_{2}\cdots \omega_{\omega }\}$.

For 32 long-gauge Bragg grating strain sensors pasted on the bridge, the bridge can be divided into 32 long-gauge elements, with each element producing a macro-strain, including macro-static strain and macro-dynamic strain. The data of two types are mixed, and the above-mentioned wavelet transform de-noising and reconstruction technology can be used for separating the macro-static strain and macro-dynamic strain. The long-gauge elements calculated using the cross-correlation function will increase by the square of the number and is termed the cross-correlation long-gauge elements, that is, there will be 1024 cross-correlation long-gauge elements. Based on the initial element, each element superimposed by the total number of elements is called the cross-correlation long-gauge element group. In order to better display the damage location effect, 1024 cross-correlation element vectors were presented in the form of matrix coordinates of 32 rows and 32 columns, as expressed in Matrix (12). When a certain element is damaged, the coordinate of the corresponding matrix diagonal position changes abruptly.

(12) $$\left[\begin{array}{ccc} \left(1,1\right) & \cdots & \left(1,32\right)\\ \vdots & \ddots & \vdots \\ \left(32,1\right) & \cdots & \left(32,32\right) \end{array}\right]$$

**Figure 3:**
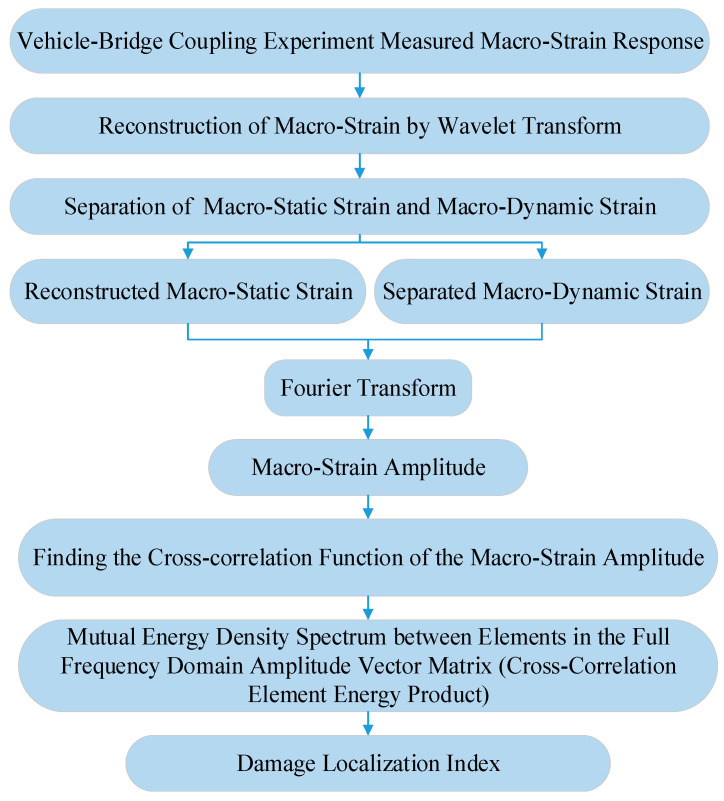
Damage localization.

The above damage localization method is summarized as shown in Figure 3.

## 4. Experimental Analysis

**Figure 4:**
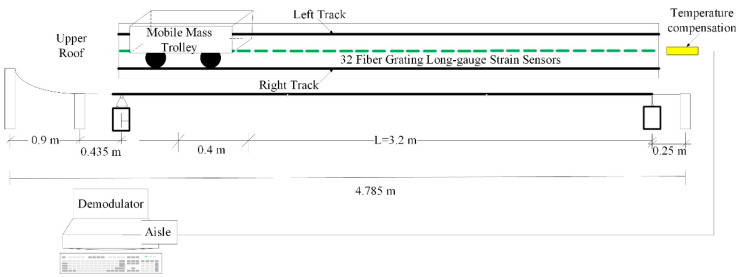
Vehicle–bridge experimental model and sensor layout diagram.

**Figure 5:**
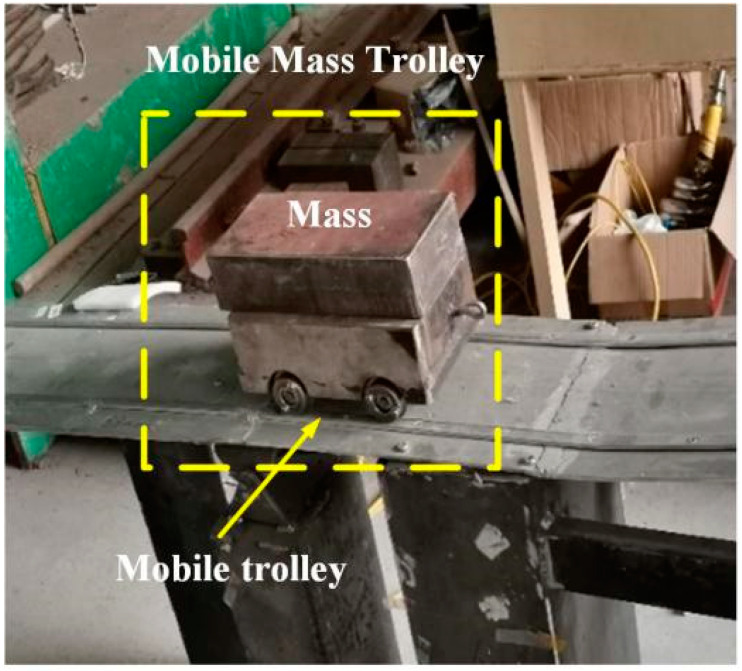
Mobile trolley.

**Figure 6:**
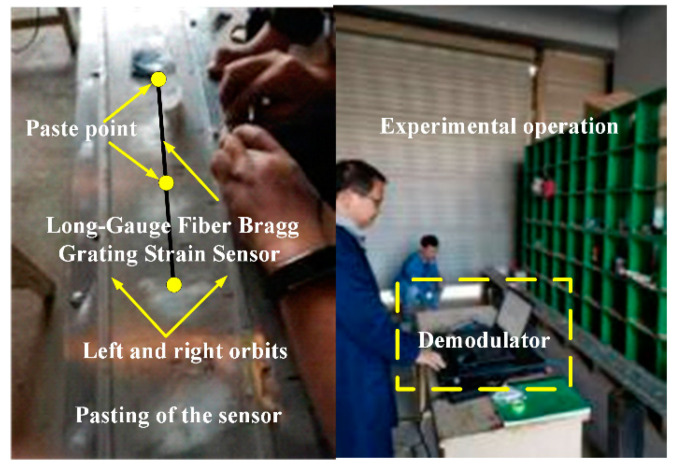
Sensor installation and experimental operation.

The span of common small and medium bridges is 24 m. According to the actual bridge, an aluminum box-type simply supported girder bridge was designed by the scale of 1:7.5. The modulus of elasticity was set to 69 GPa, the Poisson’s ratio was 0.33, the density was 2700 kg/m^3^, the dead weight was 13.07 kg, and the first-order natural vibration frequency was 16.23 Hz. Thirty-two long-gauge fiber Bragg grating strain sensors were pasted along the span of the upper roof of the beam to measure the compressive strain (taking the absolute strain value). Subsequently, the beam was divided into 32 long-gauge elements of 10 cm each. A temperature compensation sensor of the same type was connected in series. After the bonding was completed, the wavelength of the sensor was calibrated, and the wavelength of 33 strain sensors with long gauge at the same time was recorded as the initial value. Figure 4 shows the vehicle–bridge experimental model and sensor layout. The self-weight of the mobile trolley was 8 kg, and its mass could be varied by adding weight, as shown in Figure 5. Table 1 lists the damage condition settings. Figure 6 shows the experimental operation [33,34,35].

### 4.1. Reconstruction and Separation of Macro-Strain by Wavelet Transform

**Figure 7:**
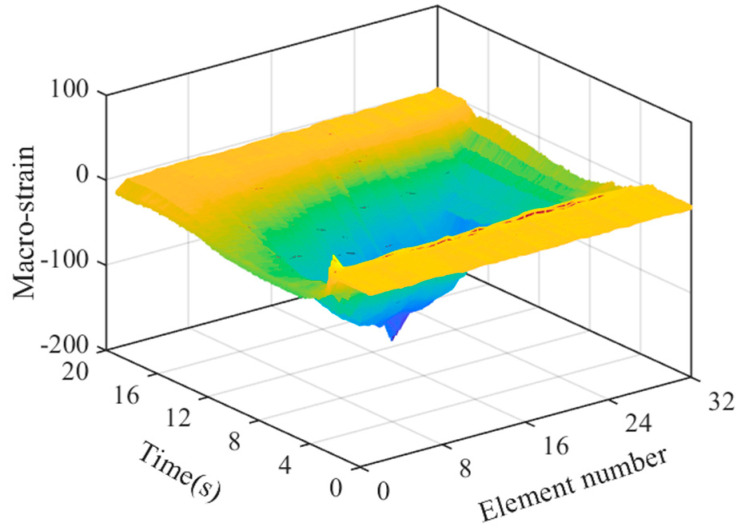
Overall strain diagram.

**Figure 8:**
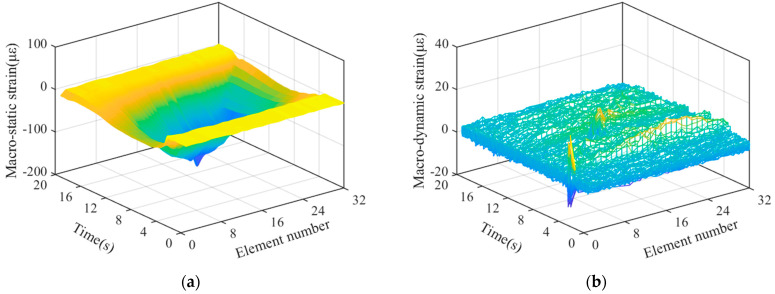
Strain reconstruction and separation under vehicle-bridge coupling; (**a**) Static strain time history diagram; (**b**) Dynamic strain time history diagram.

After sorting the macro-strain data obtained from the vehicle–bridge coupling experiment, the overall strain diagram of the moving vehicle while passing through the bridge was obtained, as shown in Figure 7. The wavelet basis function mentioned above was used for noise reduction and reconstruction to obtain the time history curve of static strain generated by the moving vehicle-mounted action on the bridge, as shown in Figure 8a. Strain separation was then performed to obtain the macro-dynamic strain curve generated by the vehicle–bridge coupling action, as shown in Figure 8b.

In the case of vehicle–bridge coupling, a time history curve of static strain is generated by the weight of the vehicle, which is not easily affected by the external environment and is suitable for quantitative damage identification. However, a macro-dynamic strain is generated by vehicle–bridge coupling, which has a small value and is easily affected by the external noise environment, making it suitable for damage localization and identification. Therefore, in this study, damage localization is performed on the basis of the damage index and time history curve of the macro-dynamic strain.

**Figure 9:**
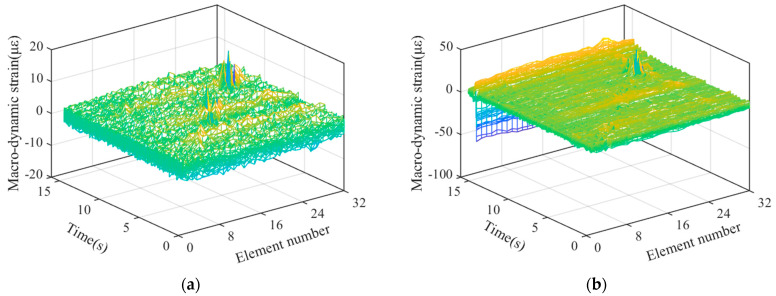
Macro-dynamic strain under damage condition for vehicle-to-bridge mass ratios; (**a**) 1.22; (**b**) 3.14.

**Figure 10:**
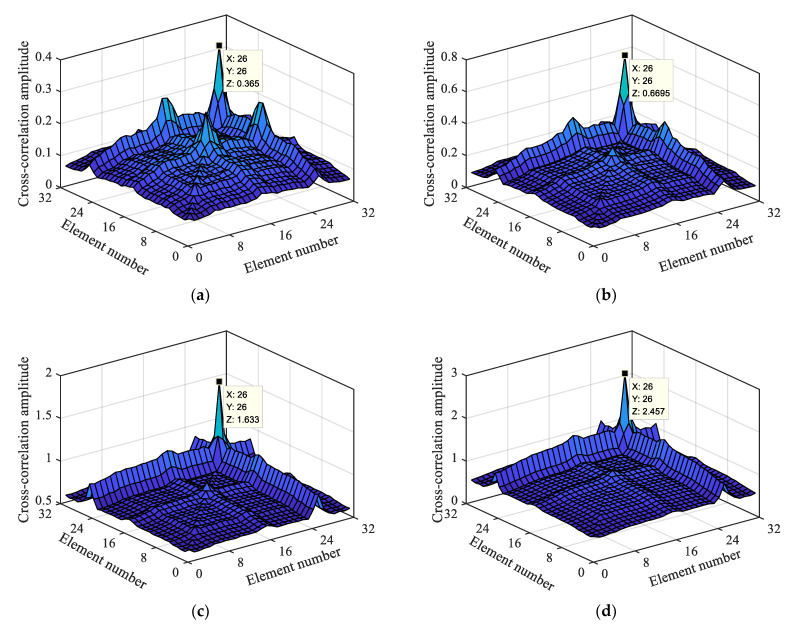
Macro-dynamic strain cross-correlation based on damage level of 2 with different vehicle-to-bridge mass ratio; (**a**) 1.22; (**b**) 1.84; (**c**) 2.45; (**d**) 3.14.

Illustration: Based on the change in the wave peak, 32 strain curves generated by the force of 32 long-gauge elements in the mid-span circuit of the upper roof of the box girder as shown in Figure 9 and Figure 10.

### 4.2. Damage Localization and Identification

Based on the separated and reconstructed macro-dynamic strain data, this section focuses on analyzing the impacts of damage degree, vehicle-to-bridge mass ratio, multiple damage locations, and other factors on the damage localization index.

#### 4.2.1. Vehicle-to-Bridge Mass Ratio

When the vehicle-to-bridge mass ratio is high, the evident vehicle–bridge coupling effect generates a macro-dynamic strain. Therefore, it is of great significance to explore the influence of the vehicle-to-bridge mass ratio on the damage localization index. Therefore, we conducted a macro-dynamic strain analysis during the actual vehicle-mounted action period; this section presents the results. Figure 9a,b show the macro-dynamic strains under the second-order damage condition with vehicle-to-bridge mass ratios of 1.22 and 3.14, respectively.

Illustration: The figures show 32 macro-dynamic strain curves indicated by different colors, which are 32 long-gauge elements in the middle span line of the upper roof.

Figure 9 shows that the macro-dynamic strain data of the 32 long-gauge elements fluctuate around 0. The vibration is most intense at 7.5 s, 10–13 s, and 14–15 s. The actual movement time of the mobile trolley is 15 s, and the trolley is controlled by a traction motor. Its speed is set to 0.2 m/s, and it takes 16 s to complete the entire process. Therefore, during the movement process of the mobile trolley, there is a condition of variable speed, that is, the 32 long-gauge elements are affected by non-stationary excitation (the process of shifting the mobile trolley is equivalent to the impact of non-stationary excitation on the bridge, making the macro-strain data more complicated). Through the analysis of the actual damage location, the mobile trolley was found to reach the middle span of the bridge at 7.5 s, reach the damage location in approximately 10–13 s, and completely leave the bridge after 15 s. The vehicle-to-bridge mass ratios of the two types have evident and sudden oscillations at 7.5 s and 10–13 s, as shown in Figure 9a,b. However, all the 32 long-gauge elements vibrate at 7.5 s, whereas in the 10–13 s range, the oscillation is only of a few long-gauge elements, as indicated by the green line in the figure. Based on the analysis of the above characteristics and the calculation method of the damage localization index, the energy product of the macro-dynamic strain cross-correlation element is obtained, as shown in Figure 10.

A total of 32 long-gauge elements were pasted on the top plate of the beam. Through the damage localization index calculation, we find 1024 cross-correlation long-gauge elements, including 32 cross-correlation long-gauge groups as shown in matrix (12). The meaning of this element group is that the mechanical vibration of each long-gauge element and the first long-gauge element are correlated. If the first long-gauge element is damaged, the macro-strain response generated by the force will be different from the macro-strain generated by the other long-gauge elements, and the calculation based on the damage localization index will make it different from the other elements. Figure 10 shows that the energy product of the cross-correlation element has an evident mutation in the (26, 26) cross-correlation long-gauge element group. Therefore, it is possible to locate the damage in the 26th long-gauge element from the sudden change in the figure.

As the vehicle-to-bridge mass ratio increases, the abrupt value of the energy product of the cross-correlation element also increases. Under the different vehicle-to-bridge mass ratios, the macro-dynamic strain generated by the vehicle–bridge coupling can realize damage location identification based on the damage localization index and the higher the vehicle-to-bridge mass ratio, the more evident the damage localization effect.

#### 4.2.2. Analysis of the Influence of Multiple Damage Locations

**Figure 11:**
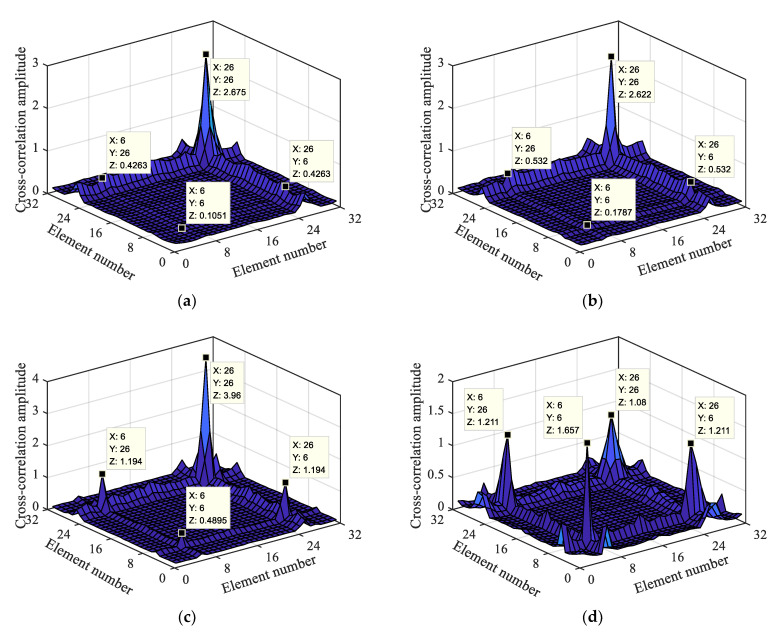
Macro-dynamic strain cross-correlation based on two damage locations with different damage levels; (**a**) Level 1 + Level 4; (**b**) Level 2 + Level 4; (**c**) Level 3 + Level 4; (**d**) Level 4 + Level 4.

Based on the reconstructed and separated macro-dynamic strain data, an identification analysis was conducted at multiple damage locations. Through the calculation of the damage localization index, the energy product values of the cross-correlation elements with different damage degrees at two damage locations were obtained, as shown in Figure 11.

Figure 11 shows mutations in the 6th and 26th cross-correlation long-gauge element groups, and the mutation rules are the same as those described in Section 4.2.1. More specifically, (6, 6) has a sudden change in the energy product at both (26, 26) and the two cross-correlation long-gauge element groups. The mutation becomes more and more evident as the damage level increases at both damage locations. However, when both damage levels reach grade four damage, they do not reach the maximum of (6, 6) and (26, 26) at the same time. Therefore, when there are multiple damages, the cross-correlation element energy product between the two damages will be weakened, which will lead to the damage localization index. The damage localization effect is reduced, particularly for minor damages. However, it is still possible to locate the damage at the 6th and 26th long-gauge elements based on the above rules. The damage localization index can realize the localization and identification of multiple damages based on the macro-dynamic strain. The greater the damage, the better the localization effect.

#### 4.2.3. Analysis of the Influence of the Damage Degree

**Figure 12:**
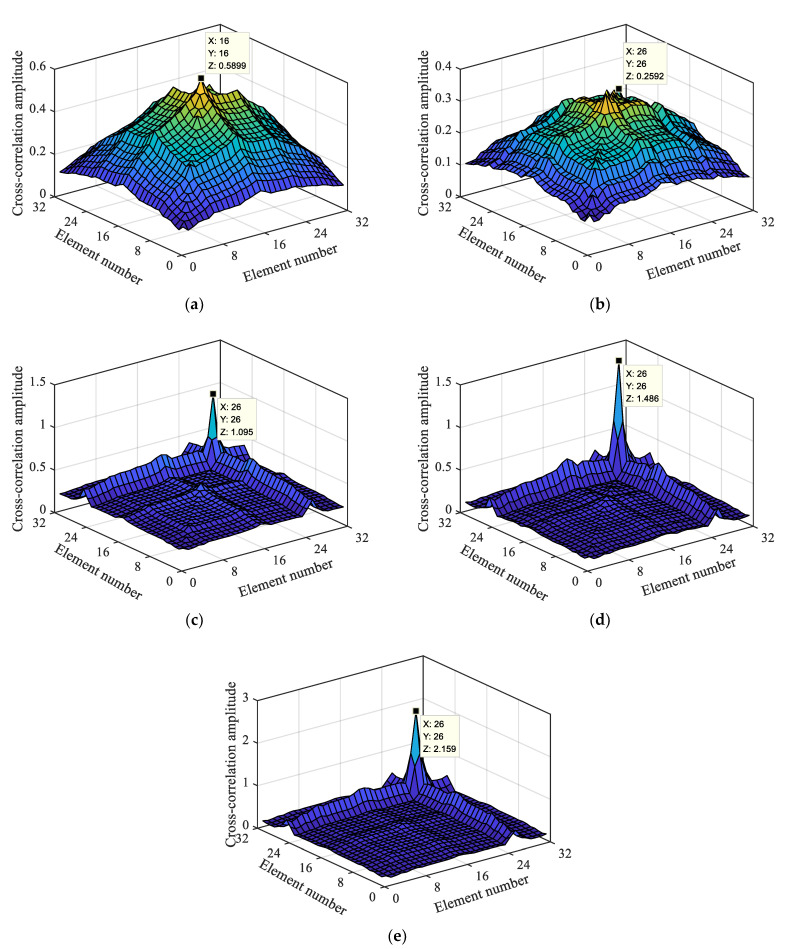
Macro-dynamic strain cross-correlation based on a single damage location with different damage levels; (**a**) Non-destructive; (**b**) Level 1; (**c**) Level 2; (**d**) Level 3; (**e**) Level 4.

To further analyze whether the damage degree would affect the positioning identification of the localization index, damage positioning research was performed with the localization index based on the macro-dynamic strain under the condition of D0–D4 for a 32 kg mobile vehicle. Through the calculation, the energy product of the macro-dynamic strain cross-correlation element was obtained, as shown in Figure 12.

Figure 12 shows the damage identification effects of the damage location index under different damage degrees. As it can be seen from Figure 12a, when the bridge is in the condition of non-destructive, the maximum value is at (16, 16), where the mid-span element of the beam is located. By comparison with Figure 12b–e, it can be seen that with the increase in damage degree, the value at (26, 26) gradually increases, and the value at (16, 16) gradually decreases. Therefore, it can be seen that damage occurred at the 26th long-gauge element, and the greater the damage degree, the more obvious the damage localization effect.

In conclusion, for the macro-dynamic strain after wavelet reconstruction and separation, the damage localization index can still be used for damage localization and identification. In terms of the vehicle–bridge mass ratio, multiple damage location, and damage degree, the higher the vehicle-to-bridge mass ratio, the greater the damage degree, the more evident the positioning effect of the damage localization index. After the reconstruction and separation, the macro-dynamic strain was not further de-noised by wavelet transform; therefore, the damage localization index based on the maximum cross-correlation number has certain anti-noise performance. Macro-dynamic strain is generated by vehicle–bridge coupling action; this confirms the feasibility of the damage localization index and identification method employed for the time-varying bridge based on the damage localization index under vehicle–bridge coupling action.

### 4.3. Experimental Error Analysis

The experimental errors mainly come from the following aspects:

1. The pre-tensioning effect of the long-gauge FBG strain sensor is weakened: As the experiment progresses, the tensile effect is increasingly weakened. Moreover, the sensor attached to the upper roof measures the compressive strain. If the pre-tensioning effect is poor, it will significantly affect the accuracy of data collection.
2. Influence of track irregularity: because of the lack of advanced processing technology, an irregularity exists in the upper roof track of the bridge. Consequently, wheel–rail collision can occur during the movement of the vehicle, producing an effect similar to percussion excitation and affecting the accuracy of strain data collection.

## 5. Conclusions and Prospects

In this study, a spatial box bridge was taken as the research object. Based on the macro-static and dynamic strain data, and the wavelet transform de-noising and reconstruction technology, a damage identification method for medium- and small-span bridges under the action of vehicle–bridge coupling was studied. The following conclusions can be drawn from the results:

1. The damage location index proposed in this paper can effectively reduce the influence of noise and other factors and accurately identify the location of subtle damage, with high accuracy. This index is not only effective for single damage location identification, but also has a good identification effect for multiple damage locations. The proposed index can provide a good reference for solving the problem that the damage index based on macro-strain can not be applied to medium- and small-span bridges due to the obvious vehicle–bridge coupling effect.
2. The proposed method in this paper has high requirements for bridge deck smoothness, moving vehicle speed and load size. The excitation caused by the collision between the wheel and the bridge deck will greatly affect the recognition effect, so it is necessary to maintain the smoothness of the bridge deck. The speed of the vehicle moving on the bridge deck is also required to be uniform, otherwise the recognition error will increase due to the excitation of variable speed. The larger the load value of the mobile vehicle is, the better the recognition effect of the damage index is. Therefore, for subtle damage, the positioning accuracy of the damage can be improved by increasing the mobile vehicle value.
3. The vehicle–bridge model designed in this study is relatively simple and lacks in-depth study under complex mobile vehicle conditions (such as moving speed and axle number). Therefore, in future research, we will consider the use of a complex vehicle–bridge coupling model and prestressed concrete beams to further improve the proposed damage identification method. The damage identification method proposed in this paper is only verified under laboratory conditions and has achieved a good damage localization effect. However, in the bridge model experiment, the scale effect is a very important factor, and this paper does not consider the influence of scale effect on the damage identification method. In the later study, the author will conduct a comprehensive analysis of the actual bridge damage and laboratory verification, in order to further analyze the impact of scale effect on the proposed damage identification method.

## Figures and Tables

**Table 1:** Damage condition table.

Damage Condition Number	Damage to the Element	Damage Width (cm)	Damage Degree (%)	
D0	0	0	0	
D1	26	0.5	Level 1 damage	
D2	26	0.5	Level 2 damage	
D3	26	5	Level 3 damage	
D4	26	5	Level 4 damage	
D5	6, 26	5, 0.5	Level 1 damage	Level 4 damage
D6	6, 26	5, 0.5	Level 2 damage	Level 4 damage
D7	6, 26	5, 5	Level 3 damage	Level 4 damage

Note: The damage degree is the percentage of damage in the entire long-gauge element. Damage width refers to the length of failure along the bridge direction at the bottom part of the box girder, which is similar to the crack. The damage location is set in the 26th or 6th long-gauge element of the lower bottom plate of the box girder. According to the damage length of the bottom plate of the box girder and the degree of damage of the web, the damage degree is comprehensively set according to the average stiffness reduction method, in which the working conditions D1 and D2 are the same damage width of the bottom plate of the box girder, and the damage of the D2 web is larger, and the overall stiffness is small. The damage is stronger, so the damage degree of D2 is greater than that of D1. D3 and D4, D5 and D6 are also set in the same way.

## Data Availability

The data presented in this study are available on request from the corresponding author.
